# Metabolic alterations and systemic inflammation in overweight/obese children with obstructive sleep apnea

**DOI:** 10.1371/journal.pone.0252353

**Published:** 2021-06-04

**Authors:** Surya Prakash Bhatt, Randeep Guleria, S. K. Kabra

**Affiliations:** 1 Department of Pulmonary, Critical Care and Sleep Medicine, All India Institute of Medical Sciences, New Delhi, India; 2 Pediatric Pulmonology Division, Department of Pediatrics, All India Institute of Medical Sciences, New Delhi, India; East Tennessee State University, UNITED STATES

## Abstract

**Aim and objective:**

Systemic inflammation has been documented in obstructive sleep apnea (OSA). However studies on childhood OSA and systemic inflammation are limited. This study aimed to determine the relation between OSA in overweight/obese children and various inflammatory markers.

**Material and methods:**

In this cross sectional study, we enrolled 247 overweight/ obese children from pediatric outpatient services. We evaluated demographic and clinical details, anthropometric parameters, body composition and estimation of inflammatory cytokines such as interleukin (IL) 6, IL-8, IL-10, IL-17, IL-18, IL-23, macrophage migration inhibitory factor (MIF), high sensitive C-reactive protein (Hs-CRP), tumor necrosis factor-alpha (TNF-α), plasminogen activator inhibitor-1 (PAI-1) and leptin levels. Overnight polysomnography was performed.

**Findings:**

A total of 247 children (190 with OSA and 57 without OSA) were enrolled. OSA was documented on polysomnography in 40% of patients. We observed significantly high values body mass index, waist circumference (WC), % body fat, fasting blood glucose (FBG), alanine transaminase (ALT), alkaline phosphate, fasting insulin and HOMA-IR in children with OSA. Inflammatory markers IL-6, IL-8, IL-17, IL-18, MIF, Hs CRP, TNF- α, PAI-1, and leptin levels were significantly higher in OSA patients (p<0.05). There was strong positive correlation of IL-6, IL-8, IL-17, IL-23, MIF, Hs CRP, TNF-A, PAI-1 and leptin with BMI, % body fat, AHI, fasting Insulin, triglyceride, FBG, WC, HOMA-IR, AST and ALT.

**Conclusion:**

Children with OSA have increased obesity, insulin resistance and systemic inflammation. Further studies are require to confirm our findings and evaluate their utility in diagnosis of OSAs, assessing severity and possible interventions.

## Introduction

Childhood obstructive sleep apnea (OSA) is characterized by episodic upper airway obstruction that occurs during sleep. OSA is associated with metabolic, cardiovascular and neuropsychological disorders [1]. OSA has been estimated to affect 2–6% of all children [2, 3] and up to 59% of obese children [4]. Childhood obesity is a significant risk factor for childhood OSA [1]. In overweight/obese children, the risk of obstructive sleep apnea syndrome (OSAS) is high at 36%, and may be increased 60% if habitual snoring is present. Similarly, Kalra et al. [5] reported that 55% of morbid obese children undergoing bariatric surgery had evidence of OSAS. Genetic and family environment play an important role in childhood obesity. It has been indicated that fat distributions in children and adults have shown visceral adiposity to be strongly associated with OSA. Body fat distribution may explain the relationship between obesity and OSA in children. Therefore other independent genetic and hormonal factors may predispose obese children to develop OSA.

It has been indicated that Inflammation is associated with OSAS. Adenotonsillar hypertrophy is a most common factor for OSAS; however, the cause of the lymphoid tissue enlargement is unknown. Chronic inflammation is also indicated by an increased expression of pro-inflammatory cytokines and elevated infiltration of macrophages into adipose tissue. Studies have shown that environmental and genetic factors may be initiated an inflammatory response to tonsillar hypertrophy [6]. Regardless, there is ample evidence to show that inflammatory markers [interleukin (IL) 6, IL-8, IL-10, IL-17, IL-18, IL-23, macrophage migration inhibitory factor (MIF), high sensitive C-reactive protein (Hs CRP), tumor necrosis factor-alpha (TNF-α), plasminogen activator inhibitor-1 (PAI-1), and leptin] have been shown to be increased in obese pediatric and adult subjects with OSA [7–12].

There is paucity of data regarding sub-clinical inflammation in patients with obese and OSA in Asian Indian children. However, till date there was not a specific biomarkers investigation particularly OSA children. In this context, we hypothesized that sub-clinical inflammation may be closely associated with obese subjects with OSA. To test this hypothesis we designed this study to analyze body composition, anthropometry, metabolic profiles and inflammatory cytokines, particularly IL- 6, IL-8, IL-10, IL-17, IL-18, IL-23, MIF, Hs CRP, TNF-α, PAI-1 leptin levels in children with and without OSA.

## Methodology

### Identification of the target population

A screening of school children was carried out between October 2014 to January 2019. A total of 247 non-diabetic overweight/ obese children were eligible for enrollment in study. 247 overweight/obese subjects [190 with OSA (cases) and 57 without OSA (controls)] included for analysis.

The study was approved by the institutional ethics committee from All India Institute of Medical Sciences, New Delhi, India and written informed consent was obtained from each participants and parents. Subjects with known type 2 diabetes mellitus (T2DM), cardiovascular disease, presence of other liver diseases (alcoholic liver disease, hepatitis virus infection, autoimmune hepatitis, primary biliary cirrhosis obstruction, drug-induced liver damage etc.), severe chronic lung disease, patients having mechanical upper airway obstruction (conformed by ENT consultant), severe end organ damage, human immunodeficiency virus (HIV) infection and other diseases like hypo and hyperthyroidism were excluded from the study. After taking consent, a detailed questionnaire was filled and eligible participants who were willing to participate in the study underwent overnight polysomnography for the diagnosis of OSA.

### Clinical and anthropometric and body composition measurements

The participants/parents were asked to complete the questionnaires on demographic characteristics, clinical profiles, personal and family medical history. Medical history was recorded with special reference to history of snoring, daytime sleepiness, any other sleep related disturbance, history of any chronic disease and of any major medical illnesses. Phenotypic markers (double chin, acanthosis nigricans, buffalo hump, skin tags, xanthelasma and arcus) were measured. Height, weight, BMI, waist circumference (WC), hip circumference (HC), waist hip ratio (WHR), mid arm circumference (MAC), mid-thigh circumference (MTC), neck circumference (NC) and skinfold thickness at 6 sites (triceps, biceps, anterior axillary, suprailiac, subscapular and lateral thoracic) were measured according to standard protocols [13]. *Body mass Index (*BMI) was calculated by using formula weight (kg)/height (m^2^). According to international obesity task force, 2015 “*Asian Cutoffs*” to define underweight (BMI < 18.5 kg/m^2^), normal weight (18.5–22.9 kg/m^2^), overweight (BMI 23–26.99kg/m^2^) and obese (BMI ≥ 27kg/ m^2^). All subjects were carefully examined physically. A four-point bioelectrical impedance apparatus (*Tanita TBF 300*, *TANITA Corp*., *Tokyo*, *Japan*), validated for Asian children and adolescents [14], was used to measure the % body fat, body fat (kg), total body water (kg) and lean body mass.

### Biochemical investigations

Five milliliter of fasting blood sample was drawn from all the enrolled subjects for following investigations.

#### Lipid profile and liver function test

The estimation of fasting blood glucose (FBG) was performed after 12 hour overnight fast with the help of commercially available kit (Randox lab ltd, United Kingdom). Estimations for total cholesterol (TC), serum triglycerides (TG), high-density lipoprotein cholesterol (HDL-C), very low-density lipoprotein cholesterol (VLDL-C), aspartate aminotransferase (AST), alkaline phosphate (ALP) and alanine aminotransferase (ALT) levels were done as previously described [13].

#### Fasting insulin and homoeostasis modal assessment for insulin resistance (HOMA)

Fasting insulin levels were measured using commercially available radioimmunoassay kits (*Immunotech*, *France*) [13]. Overall, for all the parameters the intra and inter-assay percentage coefficient and coefficient of variation were <2.0%, 1.8% and <4%, respectively.

#### Assessments of inflammatory biomarkers

Levels of hs-CRP and TNF-α was measured as previously described [15, 16]. Quantitative measurements of leptin, MIF and IL-8, PIA-1 levels were made using an automated chemiluminescence analyzer (Immulite 1000, DPC) with reagents from the same manufacturer. Levels of leptin, IL- 6, IL-10, IL-17, IL-18 and IL-23 were measured using enzyme-linked immunosorbent assay (ELISA) kit (Linco *Research Inc*., *USA*). The intra and inter-assay coefficient of IL- 6, IL-8, IL-10, IL-17, IL-18, IL-23, MIF,Hs CRP, TNF-α, PAI-1 and leptin were 3.0%, 1.9%, 2.1%, 3.2%, 1.8%, 2.6%, 3.5%, 2%, 1.97%,2.34%, 4.5 and 4%, 2.1%,3.3%,1.8%, 2.2%,3.4%, 1.8%, 2.3%, 3.5%, 3.0%, respectively.

### Polysomnography profile

All patients were called for sleep study at 8.00 pm and were attached to Alice 3 infant and pediatric computerized polysomnography system using the various leads and devices through standard gold cup electrodes. All patients underwent overnight digital polysomnography (*Medi palm; Braebon Medical Corp*., *Canada*) and classified according to apnea hypopnea index (AHI). The various parameters monitored were electrooculogram, electroencephalogram, electromyogram, an electrocardiogram and airflow, chest and abdominal efforts and arterial oxyhemoglobin saturation by pulse oximeter. At least 6 hours of polysomnography was considered as a complete study. The data recorded on the computer was manually scored for sleep stages, apneas and hypopneas. The recordings were analyzed with 60-second epoch, and sleep stages were scored according to the standard criteria [17]. Subjects with an AHI < 1/hour were assigned as not having OSA and subjects with an AHI ≥1/hour were diagnosed to have OSA. Subjects having OSA were further classified as mild (AHI 1–4.9/h), moderate (AHI 5-10/h) and severe (AHI ≥ 10/h).

### Statistical analysis

Data were entered in an Excel spreadsheet (Microsoft Corp, Washington, USA). The distribution of clinical, biochemical, anthropometry and body composition parameters was confirmed for approximate normality. All enrolled subjects were divided between two groups, with and without OSAs. Both the groups were compared for demographic, anthropometry, body composition and inflammatory markers. Categorical data were analyzed by Chi-squared test, with Fisher correction when appropriate, and expressed as absolute number (%). Continuous variables were expressed as the mean ± standard deviation to summarize the variables. All continuous values were performed using the Z score method. Pearson’s correlation coefficient and significance of ‘r’ was used to compare between IL- 6, IL-8, IL-10, IL-17, IL-18, IL-23, MIF, Hs-CRP, TNF-α, PAI-1 and leptin and other variables with adjustment for age and sex. Difference in levels of inflammatory markers in children with or without OSA was assessed using Univariate and multivariate analysis. All analyses were conducted using SPSS software for Windows, version 13.0 (Chicago, USA). Values of p < 0.05 were considered statistically significant.

## Results

### Demographic, clinical, anthropometric and body composition profiles

Demographic, clinical, anthropometric, body composition profiles were shown is Table 1. The total number of subjects were 2 47 (190 with OSA and 57 without OSA). Mean age: 10.71± 3.00 years and 11.87±2.66 years, respectively. Double chin was significantly increased in OSA children (p = 0.008). No significant difference in personal and family medical history in OSA and control group. Mean values of weight (p = 0.003), BMI (p = 0.04), fat mass (kg) (p = 0.04), % body fat (p = 0.02), WC (p = 0.05) and anterior-axillary (p = 0.05) was significantly higher in OSA as compared healthy controls. Further, Height (p = 0.01) and lean body mass (kg) (p = 0.05) was significantly increased in controls.

**Table 1:** Demographic, clinical, anthropometric and body composition profiles.

Variables	Without OSA	With OSA	P value
	(n = 57)	(n = 190)	
Age (Years)	11.87 ± 2.66	10.71 ± 3.00	0.09
**Sex**			
Males, n (%)	21(65.15)	92 (69.00)	0.14
Females, n (%)	11 (34.85)	41 (31.00)	
Birth weight (kg)	2.98 ± 0.41	2.84 ± 0.72	0.4
**Body composition**			
Height (cm)	151.61 ± 15.19	146.77 ± 17.26	**0.05**
Weight (kg)	58.4± 19.81	62.0 ± 10.68	**0.003**
BMI (kg/m^2^)	27.4 ± 4.88	27.1 ± 6.53	**0.04**
BMI Z Score	1.89±0.98	2.15±0.91	**0.02**
Fat mass (Kg)	23.46 ± 9.72	29.3 ± 55.91	**0.05**
Lean body mass (kg)	59.10 ± 82.54	45.8.12 ± 55.86	**0.05**
Total Body Water (kg)	32.9 ± 8.25	28.75 ± 8.65	0.06
% body fat	32.08 ± 7.80	38.86 ± 11.99	**0.02**
**Circumferences (cm)**			
Waist	84.52 ± 13.86	92.52 ± 10.21	**0.05**
Hip	92.39 ± 18.19	97.03 ± 15.13	**0.05**
Waist hip ratio	0.91±0.76	0.95±0.66	**0.04**
Mid-thigh	47.63 ± 9.49	46.58 ± 10.76	0.33
Mid arm	24.76 ± 4.36	24.44 ± 6.00	0.75
Neck	33.15 ± 10.95	35.03 ± 9.86	0.35
**Skinfolds (mm)**			
Biceps	18.54 ± 6.21	17.70 ± 7.02	0.51
Triceps	23.62 ± 7.25	22.95 ± 7.73	0.64
Subscapular	29.7 ± 7.16	30.90 ± 8.69	0.43
Anterior axillary	19.21 ± 6.81	21.60 ± 10.73	0.8
Suprailiac	34 ± 8.89	35.65 ± 8.70	0.35
Thigh	30.80 ± 9.58	31.11 ± 8.55	0.88
Lateral thoracic	25.92 ± 7.69	25.26 ± 7.70	0.67
Calf	24.32 ± 7.66	21.70 ± 8.90	0.17

All values are given as mean± standard deviation; n, number of subjects. P value is <0.05 is statistically significant. BMI, body mass index.

### Polysomnography finding

A polysomnography profile was shown in Table 2. Mean AHI (p = 0.001), AHI-NERM (p<0.0001), total events (p<0.0001), total events NERM (p<0.0001), total events REM (p<0.0001) and stage N3 (p = 0.04) was significantly increase in cases as compared to controls. There was no significant difference between snoring (present and past), arousal in night at present and past, daytime sleeping schedule (present and past), nasal congestion (present and past), sleep efficiency and sleep latency in cases as compared controls.

**Table 2:** Polysomnography profile.

Polysomnography variables	Without OSA (n = 57)	With OSA (n = 190)	P value
Snoring present, n (%)	10(30)	90(68)	0.073
Snoring past, n (%)	3 (9)	15(11)	0.785
Arousal in night present, n (%)	6 (17.5)	32 (23.9)	0.407
Arousal in night past, n (%)	2(5.6)	15(11.1)	0.348
Day time sleepiness present, n (%)	2 (5.5)	7(5.2)	0.402
Day time sleepiness past, n (%)	1 (3)	5 (4)	0.348
Nasal congestion/obstruction present, n (%)	3 (9)	32 (24)	0.485
Nasal congestion/obstruction past, n (%)	4(7.5)	3(8.1)	0.922
Apnea hypopnea Index (events/hour)	1.88±1.11	13.30±6.55	**0.001**
Total sleep time (minutes)	393.26 ± 54.28	408.42± 44.73	0.148
Sleep efficiency (%)	85.94 ± 6.62	89.36 ± 5.84	0.733
Sleep latency (minutes)	17.67 ± 12.95	13.47 ± 9.29	0.324
R Latency (minutes)	181.83 ± 108.12	20.16 ± 95.90	0.18
Wake after sleep onset (minutes)	47.63± 22.43	33.43 ± 25.11	**0.03**
Wake during sleep (minutes)	44.62± 22.81	30.60 ± 22.82	**0.03**
Total wake time (minutes)	65.31 ± 29.86	46.68 ± 36.57	**0.02**
Apnea hypopnea (NERM)	0.48 ± 1.11	7.0 ± 10.69	**<0.0001**
Total events (A+H+RERA) total	0.68 ± 1.13	8.45 ± 10.82	**<0.0001**
Total events (A+H+RERA) NERM	3.5± 1.09	16.3± 10.63	**<0.0001**
Total events (A+H+RERA) REM	3.8± 4.55	17.6 ± 23.85	**<0.0001**
Stage N1 (minute)	80.93 ± 44.54	77.28 ± 52.72	0.693
Stage N2 (minute)	169.9 ± 55.54	191.89 ± 62.61	0.627
Stage N3 (minute)	98.8 ± 29.80	117.7± 44.78	0.98
Stage R (minute)	26.55 ± 14.15	24.85 ± 10.62	0.489
Stage N1, %	20.73 ± 10.47	15.1 ± 11.90	0.417
Stage N2, %	27.31 ± 11.44	26.68 ± 12.38	0.869
Stage N3, %	69.730 ± 8.04	50.38 ± 11.08	0.02
Stage R, %	6.59 ± 3.57	5.96 ± 2.54	0.3
N1 latencies (minute)	2.70 ± 14.74	2.8 ± 23.41	0.709
N2 latencies (minute)	9.87 ± 18.82	13.64 ± 18.70	0.372
N3 latencies (minute)	51.39 ± 43.35	37.25 ± 34.41	0.424
R latencies (minute)	180.75 ± 107.85	202. 08 ± 95.56	0.212

All values are given as mean± standard deviation; n, number of subjects.

### Biochemical profiles

The biochemical profiles are presented in Table 3. Significantly higher values of FBG (p = 0.003), ALT (p = 0.03), ALP (p = 0.04), fasting insulin (p = 0.03) and HOMA-IR (p = 0.03) were observed in cases than in controls. Further, significantly higher values of serum potassium (p = 0.02) and creatinine (p = 0.004) were recorded in controls.

**Table 3:** Biochemical profiles.

Variables	Without OSA	With OSA	P value
	(n = 57)	(n = 190)	
**Lipid Profile (mg/dl)**			
Fasting blood glucose	93.7.69 ± 20.68	92.17 ± 23.35	0.3
Total cholesterol	160.23 ± 35.39	151.68 ± 26.83	0.34
Serum triglycerides	175.55 ± 125.09	106.13 ± 54.87	0.231
HDL-C	45.6 ± 1.97	35.87 ± 6.85	0.092
VLDL-C	35.21 ± 25.15	22.63 ± 11.37	0.284
**Insulin markers**			
Fating insulin (μU/ml)	8.2±3.2	10.4±2.3	**0.03**
HOMA-IR	1.7±0.8	2.8±0.98	**0.04**
**Liver enzymes (IU)**			
ALT	26.58 ± 17.35	39.41 ± 34.39	**0.003**
AST	27.5 ± 12.68	33.20 ± 16.70	0.09
ALP	496.51 ± 204.34	589.59 ± 247.98	**0.04**
**Other investigations**			
Albumin (gm %)	4.74 ± 0.33	4.77 ± 0.45	0.792
Bilirubin conjugated (mg %)	0.5666 ± 1.12	0.33333 ± .24	0.715
Total Protein (gm %)	7.1063 ± 0.92	7.3464 ± 7.24	0.184
Potassium (mEq/L)	135.85 ± 23.74	109.96 ± 56.86	**0.026**
Creatinine (mg %)	0.52978 ± 0.12	0.45161 ± 0.95	**0.004**
Urea (mg %)	26.92 ± 22.59	21.59 ± 5.88	0.21

All values are given as mean± standard deviation; n, number of subjects. HDL-C, high-density lipoprotein cholesterol; VLDL, very low-density lipoprotein; alanine transaminase, ALT; aspartate aminotransferase, AST, alkaline phosphate ALP, HOMA-IR, homoeostasis modal assessment for insulin resistance.

### Inflammatory markers

**Fig 1:**
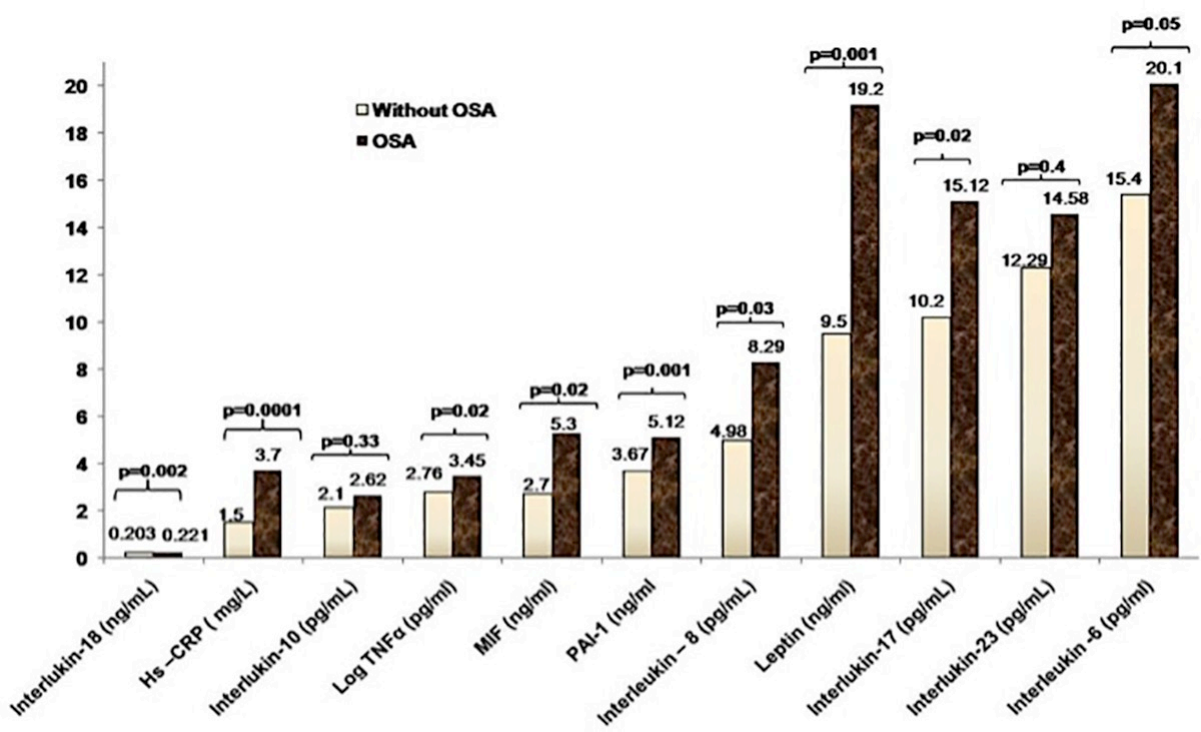
Bar graph representation of inflammatory biomarkers in subjects with and without OSA. High sensitive C-reactive Protein (Hs CRP), tumor necrosis factor alpha (TNF α), macrophage migration inhibitory factor (MIF), and plasminogen activator inhibitor -1 (PAI-1). P value <0.05 is statistically significant.

Comparison of inflammatory cytokines in OSA children with healthy controls was shown in Table 4 and Fig 1. Inflammatory markers showed significantly height levels of IL-6 (p = 0.05), IL-8 (p = 0.03), IL-17 (p = 0.02), IL-18 (p = 0.002), MIF (p = 0.02), Hs-CRP (p<0.0001), TNF-α (p = 0.02), PAI-1 (p = 0.001) and leptin (p<0.0001) in subjects with OSA as compared to healthy controls. In addition, we have indicated that inflammatory biomarkers (IL- 6, IL-8, IL-10, IL-17, IL-18, IL-23, MIF, Hs-CRP, TNF-α, PAI-1 and leptin) were significantly increased with the AHI values.

**Table 4:** Comparison of inflammatory cytokines in OSA children with healthy control.

Variables	Without OSA (n = 57)	With OSA	P value
		(n = 190)	
Interleukin -6 (pg/ml)	15.4±3.5	20.1±5.6	**0.05**
Interleukin—8 (pg/ml)	4.98 ±3.67	8.29 ±5.2	**0.034**
Interleukin-10 (pg/ml)	2.10±0.28	2.62±0.39	0.332
Interleukin-17 (pg/ml)	10.20±1.25	15.12±1.38	**0.024**
Interleukin-18 (ng/ml)	0.203±0.10	0.221±0. 109	0.2
Interleukin-23 (pg/ml)	12.29±0.73	14.58±0.75	0.47
MIF (ng/ml)	2.7±2.1	5.3±2.5	**0.026**
Hs-CRP (mg/L)	1.5±0.71	3.7±2.12	**<0.0001**
Log TNFα (pg/ml)	2.76±0.3	3.45±0.18	**0.02**
PAI-1 (ng/ml)	3.67 ± 1.51	5.12 ± 1.45	**0.001**
Leptin (ng/ml)	9.5±3.5	19.2±8.2	**<0.0001**

All values are given as mean± standard deviation. Macrophage migration inhibitory factor (MIF), high sensitive C-reactive protein (Hs CRP), Log tumor necrosis factor-alpha (TNF-α), and plasminogen activator inhibitor-1 (PAI-1).

### Association of inflammatory markers with AHI

**Fig 2:**
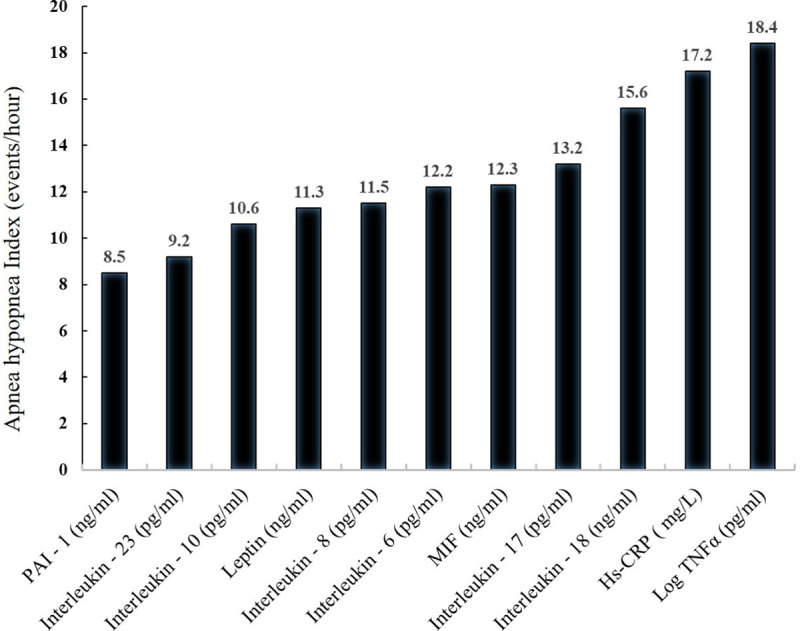
Association of inflammatory markers with AHI values.

The mean AHI was significantly higher in OSA subjects as compared to controls (p<0.05). In OSA group, all inflammatory markers were significantly increased with the AHI levels (Fig 2).

### Comparison of body composition, anthropometry, biochemical and polysomnography profiles with OSA

Mean values of BMI Z score (p = 0.05), fat mass (kg) (p = 0.05), % body fat (p = 0.03), WC (p = 0.05), FBG (p = 0.04), TC (p = 0.03), serum TG (p = 0.04), ALT (p = 0.05), AST (p = 0.04), ALK (p = 0.003), fasting insulin (p = 0.02) and HOMA-IR (p = 0.01) were significantly increased in severe OSA as compared to mild and moderate OSA (Table 5). Further, mean AHI, total events (A+H+RERA) total, total events (A+H+RERA) NERM, total events (A+H+RERA) REM and stage N1 (minute) was elevated in severe OSA subjects as compared to mild and moderate OSA (Table 6).

**Table 5:** Comparison of body composition, anthropometric and biochemical parameters with OSA.

Variables	Mild OSA	Moderate OSA (n, 56)	Severe OSA (n, 40)	p value
	(n, 94)			
**Body composition**				
Body mass index (BMI, kg/m^2^)	26.3±4.6	27.3±4.1	29.47±8.6	0.27
BMI Z score	1.94±0.12	2.03±0.43	2.24±1.04	**0.05**
Fat mass (Kg)	22.02±8.9	29.1±18.1	44.7±65.9	**0.05**
Fat Free mass (kg)	59.1±13.4	40.1±13.4	36.1±6.7	**0.02**
Total Body Water (kg)	32.1±7.5	30.3±9.6	27.26±5.6	0.20
% Body fat	31.4±8.3	34.8±6.5	40.1±15.5	**0.03**
**Circumferences (cm)**				
Waist	87.1±12.9	86.56±18.87	95.54±18.0	**0.05**
Hip	92.8±13.1	88.8±19.27	95.6±13.01	0.15
Mid-thigh	46.6±8.5	44.1±11.7	49.38±6.39	0.18
Neck	34.1±10.2	31.96±6.1	35.7±13.9	0.53
**Skinfolds (mm)**				
Biceps	19.87±7.4	16.8±4.9	19.54±8.2	0.15
Triceps	23.1±6.48	22.4±6.2	24.09±8.7	0.43
Subscapular	39.2±6.19	29.13±9.9	31.06±7.00	0.52
Antiaxillary	20.5±7.07	22.2±9.8	22.8±10.6	0.51
Suprailiac	33.4±7.8	34.7±7.3	36.09±9.5	0.62
Thigh	32.1±9.07	30.5±7.54	33.07±10.3	0.31
Lateral thoracic	25.7±6.6	24.8±6.3	26.1±8.6	0.92
Calf	24.5±7.4	21.2±7.6	25.28±9.8	0.24
**Biochemical Investigations**				
Fasting blood glucose (mg/dl)	89.4±22.5	85.7±11.1	107±32.1	**0.04**
Total cholesterol (mg/dl)	146.4±50.2	153.6±44.1	157.4±44.1	**0.03**
Serum triglycerides (mg/dl)	87.6±20.3	91.1±17.6	116.1±53.1	**0.04**
ALT (IU)	27.8±9.5	33.05±5.5	47±8.7	**0.05**
AST (IU)	28.6±4.3	29.8±6.5	38.1±10.2	**0.04**
ALK (IU)	471±200	532±171	682±110	**0.003**
Fasting serum insulin (μU/ml)	7.12±3.2	10.4±2.3	12.01±4.23	**0.02**
HOMA-IR	1.64±0.8	2.78±0.98	3.12±1.23	**0.01**

All values are given as mean± standard deviation; n, number of subjects. ALT; aspartate aminotransferase, AST, alkaline phosphate ALP, HOMA-IR, homoeostasis modal assessment for insulin resistance. P<0.05 is statically significant.

**Table 6:** Comparison of polysomnography profile with OSA.

Variables	Mild OSA (n, 94)	Moderate OSA (n, 56)	Severe OSA (n, 40)	p value
Total sleep time (minutes)	397.6±88.6	416.41±50.2	428±57.02	0.15
Sleep efficiency (%)	89±7.8	89.7±7.8	91.7±5.67	0.2
Sleep latency (minutes)	14.4±7.6	11.25±5.4	11.56±8.7	0.6
R Latency (minutes)	196.4±11.6	203.8±18.7	222±17.9	0.5
Wake after sleep onset (minutes)	35±6.6	36.01±4.2	26.4±11.2	0.2
Wake during sleep (minutes)	33.1±12.1	31±4.23	22±11.3	0.2
Total wake time (minutes)	49.4±36.2	47.03±28.6	37.9±25.9	0.2
Apnea hypopnea (NERM)	2.28±0.93	6.2±1.75	21.6±12.1	**0.0001**
Total events (A+H+RERA) total	2.66±1.36	6.16±1.86	22.8±12	**0.0001**
Total events (A+H+RERA) NERM	2.56±1.4	6.36±10.84	21.7±12.1	**0.0001**
Total events (A+H+RERA) REM	5.94±4.2	11.43±1.84	39.7±24.1	**0.0001**
Stage N1 (minute)	67.7±41.8	72.6±47.0	86.5±54.0	**0.04**
Stage N2 (minute)	188.2±60.7	184.9±54.9	210.1±69.7	0.11
Stage N3 (minute)	114.7±39.9	132.4±46.5	106.0±34.1	0.01
Stage R (minute)	25.5±15.1	26.1±9.4	25.08±16.4	0.25
Stage N1, %	17.4±10.5	17.3±10.6	20.1±12.1	0.1
Stage N2, %	46.8±10.3	44.3±11.5	48.6±12.3	0.5
Stage N3, %	29.2±9.8	32.0±10.6	25.4±9.4	0.2
Stage R, %	6.35±3.8	6.27±2.3	5.82±3.2	0.06
N1 latencies (minute)	15.8± 14.0	16.0±15.6	14.5±14.2	0.82
N2 latencies (minute)	28.2±17.3	24.2±16.1	25.8±23.1	0.9

### Correlation analysis of inflammatory biomarkers with other variables

A regression analyses was performed to demonstrate the relationships between inflammatory cytokines, anthropometric, body composition, polysomnography finding and biochemical investigations is shown in Table 7. It has been shown significant relationship (p<0.05) between pro-inflammatory cytokines and other factors, such as IL-6 with BMI (r = 0.6071), % body fat (r = 0.6138) and AHI (r = 0.5338); IL-8 with fasting Insulin (r = 0.6138); IL-10 with AHI (r = 0.657) and TG (r = 0.654); IL-17 with FBG (r = 0.598); IL-18 with WC (r = 0.682) and HOMA-IR (r = 0.689); IL-23 with % body fat (r = 0.587) and AST (r = 0.621); MIF with % body fat ((r = 0.6123), AHI (r = 0.6036) and ALT (r = 0.6156); Hs-CRP with BMI (r = 0.761); AHI (r = 0.823) and fasting insulin (r = 0.790); TNF-α with BMI (r = 0.643) and AHI (r = 0.765); PAI with % body fat (r = 0.732) and AHI (r = 0.694); leptin with BMI (r = 0.6089); AHI (r = 0.6159) and fasting insulin (r = 0.5968).

**Table 7:** Relationships among inflammatory cytokines, anthropometric, body composition, polysomnography finding and biochemical investigations in OSA patients.

	IL-6 (pg/ml)	IL-8 (pg/ml)	IL-10 (pg/ml)	IL-17 (pg/ml)	IL-18 (ng/ml)	IL-23 (pg/ml)	MIF (ng/ml)	Hs-CRP (mg/ml)	TNF-α (pg/ml)	PAI-1 (ng/ml)	Leptin (ng/ml)
**Double Chin (mm)**	0.068	0.221	-0.041	0.321	0.221	0.325	0.102	0.091	0.2987	0.3245	0.068
**BMI (kg/m^2^)**	0.6071*	0.345	-0.046	0.421	0.205	0.235	0.321	0.761*	0.643*	-0.102	0.6089*
**% Body Fat**	0.6138*	0.123	-0.104	0.265	0.421	0.587*	0.6123*	0.054	0.023	0.732*	-0.102
**Waist (cm)**	0.32	0.421	-0.379	0.421	0.682*	0.245	-0.102	0.06	0.366	-0.042	0.068
**Hip (cm)**	-0.008	0.235	0.236	0.189	-0.057	0.325	0.236	0.059	0.2456	-0.256	0.4136
**AHI (events/h)**	0.5338*	-0.102	0.657*	0.165	-0.041	0.412	0.6036*	0.823*	0.765*	0.694*	0.6159*
**FBG (mg/dL)**	-0.102	0.235	0.014	0.598*	-0.046	0.014	-0.256	0.083	-0.009	0.124	0.234
**TG (mg/dL)**	0.235	0.233	0.654*	-0.15	-0.104	-0.078	-0.102	0.099	0.659	0.237	0.214
**TC (mg/dL)**	0.368	0.112	0.215	0.165	0.256	0.208	-0.042	0.0822	0.237	0.123	0.124
**HDL (mg/dL)**	-0.005	0.145	-0.235	0.365	0.236	0.247	-0.256	0.076	0.424	0.421	-0.103
**Insulin (μU/ml)**	0.105	0.589*	0.321	0.265	0.136	0.319	-0.237	0.790*	0.370	0.068	0.5968*
**HOMA-IR**	0.061	0.247	0.236	0.365	0.689*	0.141	0.137	0.073	0.125	0.137	0.421
**ALT (IU)**	0.265	0.319	0.214	-0.196	0.165	0.236	0.6156*	0.623	0.326	-0.179	0.068
**AST (IU)**	0.356	0.221	0.082	-0.155	0.123	0.621*	0.257	-0.191	0.3568	0.237	-0.057
**ALP (IU)**	0.125	0.205	0.236	0.165	0.124	0.237	0.270	-0.176	0.127	0.158	0.124

* p<0.05. Correlation is significant at the 0.05 levels. ‘r’ is Pearson’s correlation coefficient. hs-CRP, high sensitive C reactive protein; Interleukin, IL- 6, IL-8, IL-10; macrophage migration inhibitory factor, MIF; tumor necrosis factor-alpha, TNF-α, plasminogen activator inhibitor-1,PAI-1; body mass index, BMI; waist circumference, WC; hip circumference, HP,. Apnea Hypopnea Index, AHI; fasting blood glucose, FBS; serum triglyceride, TG; total cholesterol, TC; HDL-C, high-density lipoprotein cholesterol; alanine transaminase, ALT; aspartate aminotransferase, AST, alkaline phosphate ALP, HOMA-IR, homoeostasis modal assessment for insulin resistance.

## Discussion

In this case control study, we observed that, inflammatory markers (IL-6, IL-8, IL-17, IL-18, MIF, Hs CRP, TNF- α, PAI-1 and leptin) were significantly associated with OSA as compared to those without OSAs. More importantly, there was strong positive correlation of IL-6, IL-8, IL-17, IL-23, MIF, Hs CRP, TNF-α, PAI-1 and leptin with BMI, % body fat, AHI, fasting Insulin, TG, FBG, WC, HOMA-IR, AST and ALT. In particular, such independent association and correlations are being reported for the first time.

It has been indicated that childhood obesity induces psychological, neurological, endocrine, cardiovascular, respiratory, gastrointestinal, and orthopedic abnormalities [18]. Also OSA is associated with insulin resistance, dyslipidemia, metabolic syndrome, hypertension, and inflammation in Adults. It encompasses a cluster of metabolic and cardiovascular abnormalities may play an independent role in the pathophysiology of OSAS in children as well [19]. A study from China, Li et al. [20] reported an increase in incidence of OSA and insulin levels in obese adolescents. Similarly we observed that fasting insulin and HOMA-IR levels were significantly associated and with OSA.

There is no consistent relationship between pediatric OSA and measures of body fat distribution. Verhulst *et* al. [21] investigated that the relationship between body fat distribution and OSA. He showed that WHR and total body fat has not found a clear-cut relationship between fat distribution and OSA. Another study by the same authors demonstrated that several measures of abdominal adiposity (WC, WHR, and % fat mass) correlated with oxygen desaturations associated with brief respiratory pauses, but not OSA [22]. In this study, we showed that central and abdominal obesity, body fat was significantly increased in OSA children as compared to children without OSAs.

Previous studies reported that OSA is a disease with chronic systemic inflammation, but the interaction between the different inflammatory cytokines was unclear. Systemically, Leon et al. [23] reported that the pro-inflammatory cytokines, TNF-α, IL-6, and IL-8 have been elevated in the OSA patients. Furthermore, OSA and obesity has been shown systemic inflammatory pathways, for instance serum PAI-1 levels have been significantly increased in obese OSA children [24]. In addition, hs-CRP levels have been increases in children with OSA, particularly with a threshold of an AHI > 5/hour [25]. Abdelnaby et al. [26] reported that the presence of excessive daytime sleepiness to increased TNF-α levels and the TNF-α −308G gene polymorphism. Another study has been indicated that childhood OSA was associated with higher plasma MIF, Hs CRP and fasting insulin levels [27]. We have also observed similar findings in this study, whereby TNF-α, IL-6, IL-8, MIF and PIA-1 were significantly associated with OSA was independent of the degree of obesity. Furthermore, all biomarkers altered by OSA in our subjects have been ascribed to play a patho-physiological role in cardiovascular dysfunction, thereby suggesting that OSA in obese subjects might predispose them to a more severe cardiovascular phenotype and to earlier development of cardiovascular morbidities.

The pro-inflammatory cytokines, IL-17 and IL-23, have been recently emphasized. IL-17 is a pro-inflammatory cytokine secreted by T helper 17 cells that act with other cytokines such as IL-1, TNF-α, and IL-6 to induce inflammatory diseases and play an important role in the development of autoimmunity [28]. IL-23 is a cytokine acts on memory-cluster–designation-4 (+) T cells, activates the transcription activator, and stimulates the production of interferon-gamma [29]. Huang et al. [8] reported that IL-17 and IL-23 levels were significantly elevated in pediatric OSA. These markers could be used as biomarkers of pediatric OSA. Our finding showed that IL-17 levels are significantly associated OSA, but IL-23 was significantly correlated with body fat and liver enzyme.

Leptin is a hormone with multiple functions, including regulating food intake. Tauman et al. [30] reported that leptin levels were significantly higher in sleep disorder breathing (SDB) group and also leptin levels correlate directly with BMI Z score and AHI. Another study [31] has been shown that leptin levels were higher in the SDB group. Our finding of is consistent with the previous finding. We showed that leptin levels were significantly associated with OSA and correlated with BMI, AHI and fasting insulin.

The limitations of the study include small sample size and that sample originated from north India. Further, we did not use a more accurate method of measurement of insulin sensitivity like the hyperinsulinemic euglycemic clamp technique. This was primarily due to the infrastructural constraints. Finally, longitudinal studies are warranted to study interplay between inflammation, obesity, insulin resistance, and OSA in Asian Indians.

## Conclusion

Body composition and metabolic alteration is a strong predictor of OSA. Overweight/obese children with OSA were associated with increased systemic inflammation and contribute to the relationship between inflammation, obesity and OSA. Further studies are needed to investigate the role of these biomarkers on endothelial dysfunction.
